# Speciation history of a species complex of *Primulina eburnea* (Gesneriaceae) from limestone karsts of southern China, a biodiversity hot spot

**DOI:** 10.1111/eva.12495

**Published:** 2017-06-22

**Authors:** Jing Wang, Bin Ai, Hanghui Kong, Ming Kang

**Affiliations:** ^1^ Key Laboratory of Plant Resources Conservation and Sustainable Utilization South China Botanical Garden Chinese Academy of Sciences Guangzhou China; ^2^ Southeast Asia Biodiversity Research Institute Chinese Academy of Sciences Nay Pyi Taw Myanmar

**Keywords:** allopatric speciation, biodiversity hot spots, gene flow, genetic drift, *Primulina eburnea*, species delimitation

## Abstract

Limestone karsts in southern China are characterized by high edaphic and topographic heterogeneity and host high levels of species richness and endemism. However, the evolutionary mechanisms for generating such biodiversity remain poorly understood. Here, we performed species delimitation, population genetic analyses, simulations of gene flow and analyses of floral morphological traits to infer the geographic history of speciation in a species complex of *Primulina eburnea* from limestone karsts of southern China. Using Bayesian species delimitation, we determined that there are seven distinct species that correspond well to the putative morphological species. Species tree reconstruction, Structure and Neighbor‐Net analyses all recovered four lineages in agreement with currently species geographic boundaries. High levels of genetic differentiation were observed both within and among species. Isolation–migration coalescent analysis provides evidence for significant but low gene flow among species. Approximate Bayesian computation (ABC) analysis supports a scenario of historical gene flow rather than recent contemporary gene flow for most species divergences. Finally, we found no evidence of divergent selection contributing to population differentiation of a suite of flower traits. These results support the prevalence of allopatric speciation and highlight the role of geographic isolation in the diversification process. At small geographic scales, limited hybridization occurred in the past between proximate populations but did not eliminate species boundaries. We conclude that limited gene flow might have been the predominant evolutionary force in promoting population differentiation and speciation.

## 1 INTRODUCTION

Understanding speciation is of great importance for both evolutionary and conservation biology. Traditionally, geographic isolation (allopatry) is considered the first step in species divergence, with reproductive incompatibilities eventually developed by neutral processes such as genetic drift (Dobzhansky, 1958). However, more recent theoretical and empirical studies have demonstrated that divergent natural selection across heterogeneous environments can drive population divergence and lead to speciation with or without gene flow (Coyne & Orr, 2004; Via, 2009). To understand the mechanisms underlying the processes of speciation, we need to assess the relative importance of selection, drift and gene flow in driving population and lineage divergence. Natural terrestrial islands, such as limestone karst landforms, provide a particularly interesting system for studying speciation process and the mechanisms underlying colonization, adaptation and diversification (Barbará, Martinelli, Fay, Mayo, & Lexer, 2007; Sarthou, Samadi, & Boisselier‐Dubayle, 2001). Like oceanic islands, terrestrial islands generate a range of microhabitats where ecological conditions contrast moderately to extremely from the surrounding landscape and therefore possess unique and endemic species with small population sizes and clearly defined geographic boundaries (Barbará et al., 2007; Sarthou et al., 2001). Thus, terrestrial islands act as “natural experiments” from which to study evolutionary processes of species diversity, in particularly assess the role of genetic drift and selection in population divergence and speciation.

Limestone karsts are the result of weathering of limestone rocks over time, leaving behind a terrain of dramatic peaks and caves (Ford & Williams, 2007). Across Southeast Asia and southern China, karst landscapes are disproportionately threatened by climate change and anthropogenic activities (Clements, Sodhi, Schilthuizen, & Ng, 2006). This has lead the International Union for the Conservation of Nature (IUCN) to recognize karst landscapes worldwide as being in significant need of protection (Watson, Hamilton‐Smith, Gillieson, & Kiernan, 1997). With a total area of about 400,000 km^2^, southern China is recognized as the world's type area for karst landform development in the humid tropics and subtropics (Day & Urich, 2000). Karst landforms here are typically dominated by steep cone‐shaped karst towers, caves, sinkholes and cliffs, and separated from other outcrops by lowland areas composed of different soil types. Such landscapes are characterized by diverse and extreme environmental conditions, harboring high levels of species diversity and endemism, and have been recognized as a global center of plant diversity (Davis, Heywood, & Hamilton, 1995). Substantial progress has been made in characterizing the geographic patterns of species richness and endemism in southern China (López‐Pujol, Zhang, Sun, Ying, & Ge, 2011a, 2011b; Ying, Zhang, & Boufford, 1993), yet the evolutionary mechanisms for generating the great richness of narrow endemic species in this hot spot remain poorly understood. Very few karst plant species have been the subject of phylogeographic and population genetic analyses. The few available studies revealed that allopatric speciation via geographic isolation might be the predominant mechanism in karst landforms (Chung et al., 2014; Gao, Ai, Kong, Kang, & Huang, 2015). In the allopatric mode of speciation, nonadaptive divergence, operating via genetic drift due to geographic isolation and founder effects, is expected to play a significant role in generating patterns of species diversity (Chung et al., 2014; Gao et al., 2015). However, karst areas in southern China are characterized by a high edaphic heterogeneity, with contrasting local‐scale mosaics of soil types derived from bedrock of differing lithology (e.g., granite) (Hao, Kuang, & Kang, 2015). This edaphic complexity may be a strong driver of diversification and speciation via local adaptation to specific edaphic microhabitats (i.e., specialization), as widely reported in plants (Anacker & Strauss, 2014; Anacker, Whittall, Goldberb, & Harrison, 2011; Schnitzler et al., 2011). Thus, both genetic drift and divergent selection could be strong in karst island populations. Yet, to date, no studies have reported the relative role of genetic drift, gene flow and selection as potential factors driving speciation in the karst flora in China.

*Primulina* (Gesneriaceae) is one of the species‐rich plant genera in limestone karsts of southern China, where around 170 species have been recorded, among which 150 are endemic (Möller, Wei, Wen, Clark, & Weber, 2016). *Primulina* is a monophyletic group of evergreen perennials that are widely distributed throughout the lowland karst regions of southern China and northern Vietnam. They represent a group of typical rock‐dwelling plants that have adapted to remarkably diverse habitats and niches in limestone caves and crevices. However, this genus displays a high degree of edaphic specialization, with the majority of species occurring in calcareous soils originated from limestone bedrock (i.e., calciphiles; Hao et al., 2015). As karst landforms in southern China are generally scattered and isolated as small limestone hills (i.e., terrestrial islands), most species are microendemics with narrow distribution range, often limited to a single cave or karst limestone hill system (Wei, 2010).

**Figure 1:**
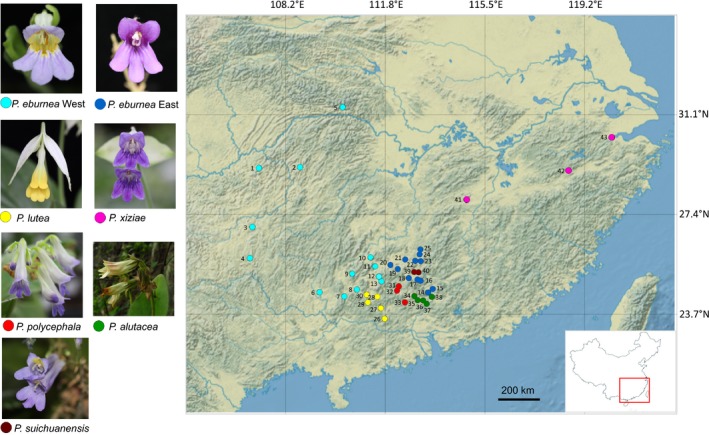
Map of the collection sites of the *Primulina eburnea* complex. Populations are color coded by species. Numbered localities correspond to population number listed in Table S1. Study region indicated by a rectangle in the lower right corner

We focus on six species that have been shown by recent phylogenetic analysis to form a monophyletic group (Kang et al., 2014), which comprises: *P. eburnea* (Hance) Mich. Möller & A. Weber, *P. lutea* (Yan Liu & Y.G. Wei) Mich. Möller & A. Weber, *P. polycephala* (W.T. Wang) Mich. Möller & A. Weber, *P. xiziae* F. Wen, Y. Wang & G.J. Hua, *P. alutacea* F. Wen, B. Pan & B.M. Wang and *P. suichuanensis* X.L., Yu & J.J. Zhou. Recently, Ning, Li, Pan, and Kang (2015) described a new rare species from southern Hunan, *P. rubribracteata* Z.L. Ning & M. Kang, which phylogenetically belongs to the species complex, but which we did not include in this study. Of the six studied species, four (*P. lutea*,* P. polycephala*,* P. xiziae* and *P. alutacea*) are calcareous soil specialists (i.e., calciphiles) and *P. suichuanensis* displays edaphic specialization on Danxia soils (after Danxia Mountain in Guangdong Province, China; they are usually red or purple in color and derived from coarse clastic rocks; Peng, Ren, & Pan, 2015), while *P. eburnea* occurs in both karst and Danxia landforms. *Primulina eburnea* has the widest distribution range of its genus, whereas the five remaining species are all narrow endemics (Figure 1). *Primulina lutea* is one of the rare yellow‐flowering species in the genus (Liu & Wei, 2004). *Primulina polycephala*,* P. alutacea* and *P. suichuanensis* occur around Nanling Mountains. *Primulina xiziae* is a newly described species morphologically similar to be *P. eburnea* (Li, Wang, Hua, & Wen, 2012). *Primulina xiziae* is the easternmost species of the genus and is found on shaded rocks in limestone hills at 70–110 m in Zhejiang province, China. This species complex shows clear geographic division and presents variation in floral and habitat specialization, making it an ideal system to study speciation of the karst flora. Previous phylogeographic analysis of this complex based on cpDNA and small numbers of microsatellites revealed strong spatial patterns of genetic structure (Gao et al., 2015). However, the cpDNA haplotypes were not sufficiently resolved to infer the geographic history of speciation. A clear understanding of speciation and diversification in karst landscapes is particularly important, as these habitats are disproportionately threatened by climate change and anthropogenic deforestation (Clements et al., 2006).

In this study, we used a panel of nuclear genes and SNPs to (i) determine the evolutionary relationships within and between species and estimate species boundaries using a coalescent‐based, Bayesian species delimitation approach, (ii) discover the patterns of genetic structure and levels of genetic diversity across species and populations, (iii) test the temporal and geographic extent of gene flow and compare evolutionary scenarios using approximate Bayesian computation (ABC) and (iv) examine whether the divergence in quantitative traits (*Q*_ST_) significantly exceeded that one detected using neutral markers (*F*_ST_) as signal of divergent selection by pairwise comparisons of *F*_ST_ and *Q*_ST_ estimates. Our main objectives are to investigate processes of speciation in the *P. eburnea* complex and evaluate the role of genetic drift, gene flow and natural selection on population divergence and speciation.

## 2 MATERIALS AND METHODS

### 2.1 Population sampling, DNA sequencing and SNP genotyping

A total of 405 individuals from 43 populations (five to 14 per population) were sampled across the geographic range of six closely related *Primulina* species, *P*. *eburnea*,* P*. *lutea*,* P*. *polycephala*,* P*. *alutacea*,* P*. *suichuanensis* and *P*. *xiziae* (Figure 1; Table S1). A previous study revealed that *P*. *eburnea* was divided into two deeply divergent phylogenetic lineages representing an eastern and a western distribution (Gao et al., 2015), so we treated these western and eastern populations as distinct species in the data analysis. In addition, one individual of *P*. *tabacum* was sampled as the out‐group in phylogenetic analysis. Total genomic DNA was extracted from dried leaves using a modified cetyltrimethylammonium bromide (CTAB) method (Doyle & Doyle, 1987).

Six single‐copy nuclear DNA (nDNA) loci developed from comparative transcriptomic data of multiple *Primulina* species (Ai et al., 2015) were amplified and sequenced (Table S2). Polymerase chain reactions (PCRs) were performed in total volume of 25 μl containing 10–40 ng DNA template, 50 mM Tris‐HCl (Takara), 1.5 mM MgCl_2_ (Takara), 0.5 mM dNTPs (Takara), 0.75 unit of Taq polymerase (ExTaq, Takara) and 2 mM of each primer in final concentration. All reactions were performed using the following temperature profile: 5 min at 94°C, 35 cycles of 30 s at 94°C, 30 s of annealing at 57°C, and 90 s at 72°C, with a final 10‐min extension at 72°C. The PCR products were sequenced directly using the BigDye Terminator Cycle Sequencing kit according to the instructions of the manufacturer (Applied Biosystems, Foster City, CA, USA) on an ABI 3730XL automated sequencer. All sequences were deposited in GenBank with accession numbers KY239620‐KY241086.

Five hundred and forty‐one SNP loci developed from the *Primulina* transcriptome (Ai et al., 2015) were genotyped for all 405 individuals on the MassARRAY platform (Sequenom, San Diego, CA). The reliability and cross‐species transferability of these loci have been validated in *P. eburnea* (Ai et al., 2015). The paired PCR and single extension primers were designed based on the flanking sequences using Sequenom Assay Design 3.1 software. The whole pipeline included PCR primer mix preparation, multiple PCR amplification, shrimp alkaline phosphatase reaction, extension reaction, resin mixture, sample spotting, mass spectrometry and genotype reading. The multiple PCR amplification was performed in a reaction volume of 5 μl containing 10 ng DNA template, 1 unit of Taq polymerase, 2 mM MgCl_2_, 0.5 mM dNTPs and 0.1 μM PCR primer mix in final concentration. The multiple PCR temperature profile was set as follows: 2 min at 95°C, 45 cycles of 30 s at 95°C, 30 s at 56°C, and 60 s at 72°C, with a final 5‐min extension at 72°C.

### 2.2 Genetic diversity

Heterozygous nuclear sequences were phased to alleles using phase v2.2.1 (Stephens, Smith, & Donnelly, 2001). Seqphase (Flot, 2010) was used to convert input and output formats for phase. phase computations were conducted with default settings (phase threshold = 90%, iteration steps = 100, thinning interval = 1, burn‐in steps = 100) and were repeated three times to ensure consistency. The program dnasp 5.10.01 (Librado & Rozas, 2009) was used to calculate the number of segregating sites (*S*), the number of haplotypes (*h*), haplotype diversity (*Hd*) and nucleotide polymorphism (π) for each population and species. Because nonneutral data may bias the following analyses, we performed multiple neutrality tests to confirm whether the loci were neutral. We calculated Tajima's *D* (Tajima, 1989) to test for departure from the standard neutral model using dnasp. To discriminate between neutral and adaptive evolution, we performed the multilocus HKA test (Hudson, Kreitman, & Aguade, 1987) using the HKA package (https://bio.cst.temple.edu/~hey). Individual runs of the HKA test were performed for the contrast between each of the seven species and the out‐group.

For SNP data, we trimmed the dataset by excluding loci with minor allele frequency (MAF) lower than 0.01 and individuals with more than 10% missing genotypes across all loci. This led to a total of 384 individuals from 43 populations with 414 loci in the data analysis. We estimated percentage of polymorphic loci (*PPL*), the number of different alleles (*A*), the number of effective alleles (*A*_*e*_), observed heterozygosity (*H*_O_), expected heterozygosity (*H*_E_) and fixation index (*F*_IS_) for each population and species using GenAlEx v6.502 (Peakall & Smouse, 2012). The population assignment, genetic differentiation, AMOVA and network clustering based on SNP data were similar as those based on nDNA sequence data.

### 2.3 Population structure and genetic clustering

We used a Bayesian clustering approach implemented in Structure v2.3.4 (Pritchard, Stephens, & Donnelly, 2000) to infer the number of clusters and to assign individuals to these clusters. We ran 10 independent replicates for each *K* value between 1 and 10, with 100,000 burn‐in MCMC steps followed by 150,000 recorded steps. The admixture model and uncorrelated allele frequencies between populations were specified for each run. We used structure harvester (Earl & vonHoldt, 2012) to summarize the results including the identification of optimal *K* through the method of Evanno, Regnaut, and Goudet (2005). Results across replicate runs were combined using the program clumpp (Jakobsson & Rosenberg, 2007) and visualized with distruct v1.1 (Rosenberg, 2004). We performed analysis of molecular variance (AMOVA) using arlequin v3.5.2.2 (Excoffier & Lischer, 2010) to calculate the respective proportion of genetic variation at three hierarchical levels: among groups, among populations within groups and within populations. The groups were defined according to species or the inferred clusters. The significances of variance components were estimated with 1,000 permutations. We also calculated genetic differentiation (*F*_ST_) between population and species using arlequin v3.5.2.2 (Excoffier & Lischer, 2010).

We further performed a phylogeographic analysis using the Neighbor‐Net method implemented in SplitsTree4 (Huson & Bryant, 2006) based on the uncorrected *p*‐distance between individuals to reveal the overall genetic pattern of relationships among populations and individuals.

### 2.4 Phylogenetic reconstruction

Prior to phylogenetic reconstruction, the best substitution model for each nDNA locus (Table S2) was determined under the Akaike information criterion as implemented in jModelTest v2.1.3 (Darriba, Taboada, Doallo, & Posada, 2012). Gene trees of the identified haplotypes were reconstructed using Bayesian inference as implemented in mrbayes v3.2.6 (Ronquist et al., 2012). The Bayesian analyses were run three times with one cold and three heated chains for ten million Markov chain Monte Carlo generations. Trees were sampled every 1,000 generations, and the first 25% generations were discarded as burn‐in. tracer v1.5 (Rambaut & Drummond, 2009) was used to check convergence and effective sample sizes of parameter estimation. We estimated the species tree of the six *Primulina* species and the population tree of the 38 populations using *beast v1.8.3 (Heled & Drummond 2010). We ran the MCMC analysis three times for 500 million generations with sampling every 50,000 generations. The relaxed clock model with an uncorrelated lognormal distribution, the constant population size and the Yule speciation tree prior were also specified in the settings. The final trees were visualized using both FigTree v1.4.2 (http://tree.bio.ed.ac.uk/software/figtree/) and DensiTree in the beast package.

### 2.5 Species delimitation

Bayes factor delimitation is a useful method that compares the marginal likelihoods of competing species delimitation hypotheses (Grummer, Bryson, & Reeder, 2014). Based on a combination of the results from population clustering and phylogenetic analyses, four alternative species delimitation models were tested against the current delimitation model (seven species): (i) a six‐species model where the *P*. *eburnea* (east) and *P*. *xiziae* were lumped together; (ii) a second six‐species model where the eastern and western populations of *P*. *eburnea* were lumped together; (iii) a five‐species model where the three species of *P*. *polycephala*,* P*. *alutacea* and *P. suichuanensis* were lumped together; and (iv) a four‐species model where the *P*. *eburnea* (east) and *P*. *xiziae* were lumped together and simultaneously, the three species of *P*. *polycephala*,* P*. *alutacea* and *P. suichuanensis* were lumped together. We used *beast v1.8.3 to estimate species trees for the above species delimitation models based on the six nDNA loci dataset. Each locus was given its own partition with the preferred substitution model, and the uncorrelated relaxed lognormal clock was specified. *beast analyses were performed for 500 million generations, logging every 50,000 generations. For each hypothesis, three *beast replicates were conducted to check convergence and effective sample sizes using tracer v1.5. Marginal likelihoods were estimated using path sampling (PS) and stepping stone (SS) methods (Baele et al., 2012; Grummer et al., 2014), with 100 path steps, a chain length of 100,000 generations and likelihoods logged every 100 generations. The four alternative models were compared against the current delimitation model, and the significantly better models were determined whether the Bayes factor value (twice the difference in marginal likelihood estimates) was larger than 10 (Kass & Raftery, 1995).

### 2.6 Estimation of gene flow

Four approaches were used to estimate spatial and temporal pattern of gene flow. Firstly, to distinguish hybridization or introgression from incomplete lineage sorting in causing phylogenetic incongruence between gene trees and species trees, we used jml v1.3.0 (Joly, 2012) to test whether the minimum distance between sequences of two species is smaller than expected under a scenario without hybridization. To do so, the observed minimum distance between sequences is compared to a null distribution obtained by simulating a scenario without hybridization. The posterior distributions of species trees, population sizes and divergence times included in the output of the *beast analyses were used as input for jml. The best substitution models for individual genes were specified in the control file, while the default values were used in other settings.

**Figure 2:**
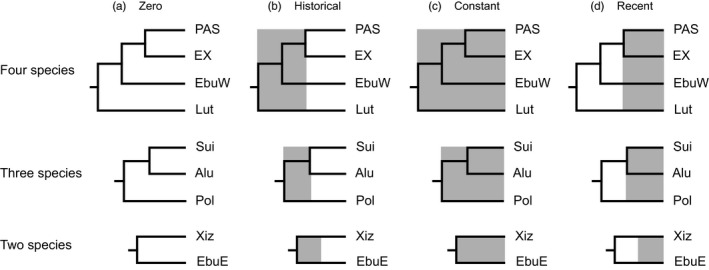
Four scenarios of gene flow (indicated by shading) simulated under the neutral coalescent and evaluated using approximate Bayesian computation from the four lineages among EubW, EX, Lut and PAS (four‐species model); three species among Pol, Alu and Sui (three‐species model) and two species between EbuE and Xiz (two‐species model). Lineage and species abbreviations are defined in Table 1

Secondly, we estimated population migration rate based on the isolation with migration (IM) model implemented in ima2p (Sethuraman & Hey, 2016). We performed IM estimation with three separate datasets: the four lineages including all species (four‐species model; Figure 2), the PAS lineage including three species of *P. polycephala*,* P. alutacea* and *P. suichuanensis* (three‐species model; Figure 2) and the EX lineage including *P. eburnea* (east) and *P. xiziae* (two‐species model; Figure 2). For each IM model, we ran analyses of 20 Markov chains with heating terms of 0.96 (‐ha) and 0.90 (‐hb), for 300,000 MCMC steps after a burn‐in of 300,000 steps. Of the nucleotide substitution models implemented in the program ima2p, HKY was the best model and thus was applied for all genes. The mutation rates were calculated based on the transcriptome‐wide divergence (Ai et al., 2015) and molecular dating results (Gao et al., 2015). Finally, we used IMfig (https://bio.cst.temple.edu/~hey/software) to generate the result figures of the IM models.

Thirdly, we used the approximate Bayesian computation approach as implemented in the program MSABC (Pavlidis, Laurent, & Stephan, 2010) to evaluate the posterior probabilities of four alternative evolutionary scenarios: zero, constant, historical or recent gene flow models (Figure 2). In the first two models, lineages evolve without gene flow or with constant gene flow throughout the divergence process. In the other two models, gene flow only occurs in or following the initial divergence stages. We ran 10^6^ simulations under each model based on our empirical priors estimated in this study or previous attempts (Ai et al., 2015; Gao et al., 2015), including locus number, sample size, sequence length, population mutation rate and divergence time. A migration rate of 1 *N*_e_m (0.5 *N*_e_m in each direction) was used because this is the maximum migration rate allowed between separate species (Hey, 2009). For the ABC analysis, we chose *F*_ST_ as the summary statistic to describe the degree of differentiation among lineages. We transformed observed sequence data into ms‐like files using the fas2 ms perl script in the MSABC package and calculated the same summary statistic. Based on the comparison between simulated and observed statistics, we used the “postpr” function in R package “abc” (Csilléry, François, & Blum, 2012) to estimate posterior probabilities, adopting the rejection method and a tolerance of 0.001.

Finally, we performed coalescent simulations in conjunction with the genealogical sorting index (gsi; Cummings, Neel, & Shaw, 2008) to determine the temporal period of gene flow, i.e., whether gene flow occurred among ancestral or extant populations. The gsi is a statistic that estimates the degree of exclusive ancestry of each delimited taxon on a scale ranging from 0 (nonexclusive) to 1 (monophyletic). Coalescent simulations were performed using mccoal (Rannala & Yang, 2003; Yang & Rannala, 2010). In our simulations, a symmetric migration matrix with the migration rate constant at 0 or 0.5 *N*_e_m in both directions was used, and divergence times and population sizes were derived from our empirical estimates (Gao et al., 2015). The four simulated scenarios included zero, historical, constant, and recent gene flow scenarios (Figure 5). Based on 10,000 simulated trees under each scenario, a gsi value was calculated for each species within each tree using the R package genealogicalSorting v0.92 (Cummings et al., 2008). An empirical gsi value was also calculated based on the inferred tree in mrbayes v3.2.6. The comparison of empirical gsi values to the simulated gsi values was used to determine the timing of migration events.

### 2.7 Analysis of floral morphological data

During March–September 2015, floral morphological data were collected from plants cultivated in a greenhouse at South China Botanical Garden (SCBG). The plants were grown in 12‐cm‐diameter pots filled with Klasmann substrate (Klasmann Deilmann, Geeste, Germany). Five floral traits, including corolla length (CL), corolla height (CH), corolla width (CW), upper petal width (PW1) and lower petal width (PW2), were measured from five flowers per individual for a total of 129 individuals across 22 populations. Data were tested for normality using Kolmogorov–Smirnov normality test, and the nonnormal distributions (i.e., PW1 and PW2) were log transformed. To assess whether divergence of measured floral traits was associated with genetic divergence, we calculated the degree of quantitative phenotypic divergence between populations as *Q*_ST_ = σ_B_^2^/(σ_B_^2 ^+ 2σ_W_^2^), where σ_B_^2^ is the variance between populations and σ_W_^2^ is the within‐population variance (Spitze, 1993). Because all morphological traits measured in this study were floral traits, and therefore, nonindependent, *Q*_ST_ values were estimated from the first two principal components (PC1 and PC2). Pairwise *Q*_ST_ values between populations were calculated for each lineage/species separately. We used the R package *QstFstComp* (Gilbert & Whitlock, 2015) to compare the mean pairwise *Q*_ST_ matrices to the mean pairwise *F*_ST_ matrix estimated from the SNP dataset. If the value of *Q*_ST_ significantly exceeds *F*_ST_, then a hypothesis of divergent selection would be supported, whereas *Q*_ST_ and *F*_ST_ values that do not differ significantly are consistent with neutral genetic differentiation (Merilä & Crnokrak, 2001).

## 3 RESULTS

### 3.1 Genetic diversity and pairwise differentiation

The six nDNA regions varied in length from 669 to 1,197 bp and totaled 5,613 bp with 803 variable sites, 532 of which were parsimony‐informative (Table S2). No recombination was evident for any of the six nuclear loci. Descriptive statistics for the nDNA loci across populations exhibited low‐to‐moderate haplotype and nucleotide diversity, with about half of the population–locus combinations (107 of 228) containing either a single or two haplotypes (Table S3). Species‐level haplotype and nucleotide diversity estimates from the nDNA dataset indicated fairly even diversity across species, with slightly lower diversity in *P. xiziae*, the easternmost species (Table 1). For the SNP dataset, similarly low levels of genetic diversity were found across populations, with mean percentage of polymorphic SNPs varying from 8.94% to 68.36% (Table S4). Again, similar levels of effective number of alleles and expected heterozygosity over all loci (*A*_e _= 1.2–1.4 and *H*_E _= .096–.256) were found across species, with slightly lower diversity in *P. xiziae* (Table 1).

**Table 1:** Summary statistics of polymorphism for the seven taxa based on six single‐copy nuclear genes and 414 SNP loci

Lineage abbreviation	Species abbreviation	Species	nDNA					SNP					
			*n*	*S*	*h*	*H*_d_	π	*n*	*PPL*	*A*	*A*_*e*_	*H*_O_	*H*_E_
EbuW	EbuW	*P. eburnea* (west)	72	47	30	.891	.0066	115	75.4	1.8	1.3	.128	.158
Lut	Lut	*P. lutea*	30	19	13	.847	.0038	50	73.9	1.7	1.4	.206	.236
PAS	Pol	*P. polycephala*	18	27	11	.898	.0051	25	53.1	1.5	1.3	.215	.160
	Alu	*P. alutacea*	24	25	15	.935	.0066	47	61.8	1.6	1.3	.176	.170
	Sui	*P. suichuanensis*	13	9	7	.859	.0028	18	48.3	1.5	1.3	.214	.156
EX	EbuE	*P. eburnea* (east)	67	45	35	.923	.0059	104	90.1	1.9	1.4	.196	.256
	Xiz	*P. xiziae*	23	12	6	.617	.0027	25	45. 9	1.5	1.2	.113	.096

*n,* number of individuals; *S*, number of segregating sites; *h*, number of haplotypes; *H*_d_, haplotype diversity; π, nucleotide diversity; *PPL*, percentage of polymorphic SNP loci; *A*, number of alleles; *A*_*e*_, effective number of alleles; *H*_O_, observed heterozygosity; *H*_E_, expected heterozygosity.

We found high genetic differentiation at both the population and species levels. An AMOVA across all populations shows that most of the variance (68% and 59.1% for nDNA and SNP datasets, respectively) is found among populations (Table 2). For the nDNA sequence data, pairwise estimates of *F*_ST_ between populations ranged from 0.073 to 1 (mean = 0.712), and all comparisons were highly significant (*P *<* *.001; Table S5). This translated into a high level of genetic differentiation among species (*F*_ST _= .204–.751; Table 3). For the SNP dataset, pairwise *F*_ST_ values between populations varied from 0 to 0.893 (mean = 0.539; Table S6). Pairwise *F*_ST_ values between species were lower than that calculated from the nDNA dataset, but highly significant (Table 3).

**Table 2:** Results of the analyses of molecular variance (AMOVA) based on nuclear DNA sequence (nDNA) and SNP datasets

Source of variation	nDNA		SNP	
	d.f.	Percentage of variation	d.f.	Percentage of variation
All populations				
Among populations	37	68.0^a^	42	59.1^a^
Within populations	209	32.0^a^	725	40.9^a^
Four lineages				
Among clades	3	28.5^a^	3	43.5^a^
Among populations within clades	34	41.9^a^	39	20.1^a^
Within populations	209	29.6^a^	725	36.4^a^
Seven species				
Among species	6	63.2^a^	6	43.6^a^
Among populations within species	31	8.3^a^	36	18.8^a^
Within populations	209	28.5^a^	725	37.6^a^

a *p *<* *.0001; *p* values were estimated based on a permutation test (1,000 randomizations).

**Table 3:** Summary of the pairwise *F*_ST_ values among *Primulina* species based on six single‐copy nuclear genes (upper triangular) and 414 SNPs (lower triangular)

	EbuW	EbuE	Lut	Pol	Alu	Sui	Xiz
EbuW		.495	.578	.531	.528	.555	.570
EbuE	.528		.611	.545	.531	.560	.365
Lut	.439	.559		.711	.624	.710	.751
Pol	.387	.393	.414		.417	.513	.702
Alu	.369	.403	.419	.115		.204	.646
Sui	.403	.376	.420	.091	.070		.739
Xiz	.602	.272	.622	.549	.516	.541	

All *F*_ST_ values are significant (*p *<* *.001). Species abbreviations are given in Table 1.

### 3.2 Phylogenetic reconstruction

**Figure 3:**
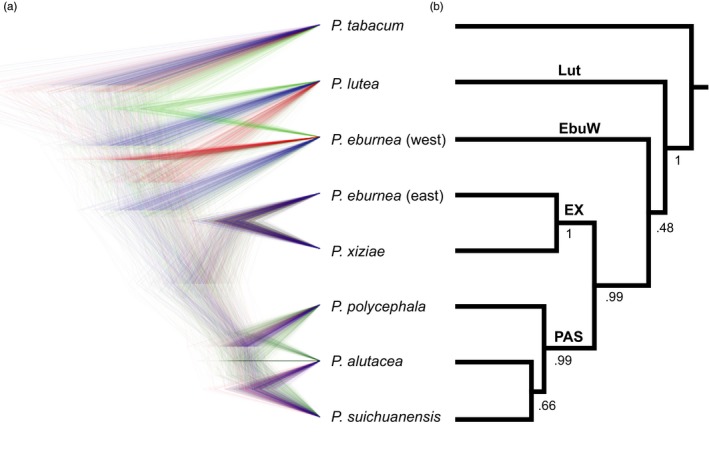
Estimated species trees from six single‐copy nuclear loci using the framework of the multispecies coalescent algorithm implemented in *beast: (a) DensiTree visualization of consensus; (b) consensus tree. Posterior probabilities are given at each node. The four recovered lineages were defined as Lut, EbuW, EX and PAS

**Figure 4:**
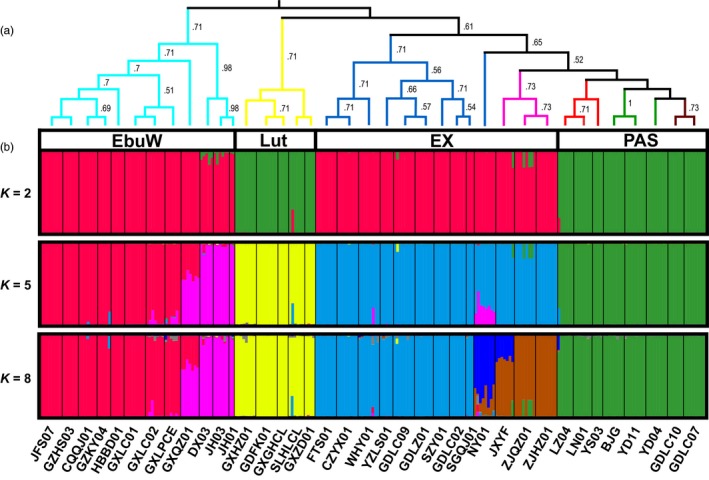
(a) Phylogenetic tree of the 38 populations based on six single‐copy nuclear loci with posterior probabilities >50 indicated on the nodes. The out‐group was trimmed. Populations are color coded by species, and the four lineages are indicated: EbuW, EX, Lut and PAS; (b) Bayesian assignment of 247 individuals using structure with *K *=* *2, 5 and 8. Each individual is represented by a vertical bar and grouped by population and species. Population names correspond to Table S1

As expected, individual gene trees were poorly resolved (Fig. S1), likely due to the limited number of variable sites in these loci. Yet, the nDNA data show clear phylogenetic signal and several generalizable patterns. *P. lutea* haplotypes formed a clade in four of six gene trees, *P. eburnea* (west) haplotypes formed a clade in three of six gene trees, haplotypes of *P. eburnea* (east) and *P. xiziae* formed a clade five of six gene trees and haplotypes of *P. polycephala*,* P. alutacea* and *P. suichuanensis* formed a clade in three of six gene trees. Accordingly, the Bayesian species tree estimated using *beast for the multilocus dataset recovered four lineages (Figure 3) in general agreement with currently species geographic boundaries. Populations of the same species always phylogenetically clustered with high support, except for two populations (NY01 of *P. eburnea* (east) and YD04 of *P. alutacea*) (Figure 4). Support for the four retrieved lineages is high in the species tree; however, it is very low (0.48) for the node splitting *P. eburnea* (west) and lineages of PAS and EX, indicating significant ambiguity in this topology.

### 3.3 Genetic clustering

**Figure 5:**
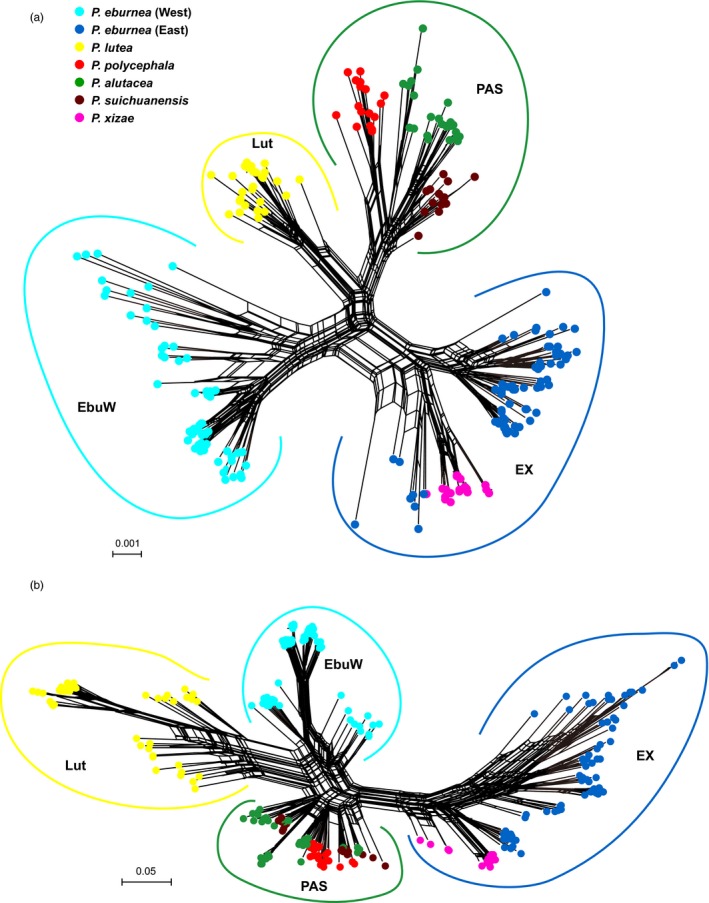
Neighbor‐Net network showing genetic relatedness based on uncorrected p‐distances from six single‐copy nuclear loci (a) and 414 SNP markers (b). Individuals are colored by species. Four lineages are indicated: EbuW, EX, Lut and PAS

The Neighbor‐Net networks (Figure 5) based on nDNA and SNP datasets were largely congruent and mirror the general pattern evident in the phylogenetic analyses. In both networks, four major clusters corresponding to the four phylogenetic lineages were identified: (i) EbuW, the western *P. eburnea* populations; (ii) Lut, the *P. lutea* populations; (iii) PAS, all populations of *P. polycephala*,* P. alutacea* and *P. suichuanensis*; and (iv) EX, the populations of eastern *P. eburnea* and *P. xiziae*. Almost all individuals cluster according to respective species, and in most case species are supported by strong split. Exceptions were individuals of *P. polycephala*,* P. alutacea* and *P. suichuanensis*, which formed a mixture cluster in the SNP dataset; individuals of eastern *P. eburnea* populations (NY01 and CZGY01) form a sub‐cluster with *P. xiziae*.

Structure analyses based on nDNA dataset inferred *K *=* *5 as the optimal number of groups. The supported major groups are generally congruent with genetic clusters generated with Neighbor‐Net networks, but the western *P. eburnea* cluster was further divided into two groups. On the other hand, the model with *K *=* *8 was also highly supported under the Evanno method, with a delta *K* value (Fig. S2) similar to the preferred *K *=* *5. At *K *=* *8, the cluster of the eastern *P. eburnea* and *P. xiziae* were further divided into three groups: the eastern *P. eburnea* populations excluding the NY01, the NY01 and *P. xiziae*. However, the clustering at *K *=* *8 is essentially identical to *K *=* *7, because no individuals are completely assigned to a new group (Figure 4). All of the main genetic clusters were geographically coherent, and only populations GXQZ01, NY01 and JXGF01 showed admixture above a cutoff level of 22%.

For the SNP dataset, Structure analyses revealed fewer distinct groups, with the inferred *K *=* *2 and *K *=* *4 as the optimal and sub‐optimal numbers of groups, respectively. However, a population grouping of *K *=* *4 was more biologically informative, because the four population groups are generally corresponded to the four phylogenetic lineages identified with the nDNA dataset. At *K *=* *4, the first group consisted of nine western *P. eburnea* populations. The second group was composed of the five *P. lutea* populations, and the third group consisted of the eastern *P. eburnea* populations and the three *P. xiziae* populations. The fourth group included all populations of *P. polycephala*,* P. alutacea* and *P. suichuanensis*, plus four populations (GXQZ01, DX03, JH01, JH03) of western *P. eburnea* (Fig. S3).

### 3.4 Species delimitation

Bayes factor delimitation marginal likelihood values for alternative species delimitation models are listed in Table 4. The favored result is a seven‐species model that corresponds to the morphological species description, with exception for the recognition of western and eastern clades of *P. eburnea* as two distinct species. In fact, the “six‐species” model that grouped western and eastern populations of *P. eburnea* into a single species was the lowest ranked model, indicating that this species needs taxonomic revision.

**Table 4:** Summary of Bayes factor delimitation of species (BFD) analyses

Model^a^	Path sampling		Stepping stone sampling	
	Likelihood	BF	Likelihood	BF
Seven‐species	−35,858	0	−39,888	0
Six‐species (a)	−43,380	15,044	−52,385	24,994
Six‐species (b)	−38,432	5,148	−44,575	9,374
Five‐species	−38,701	5,686	−42,499	5,222
Four‐species	−39,464	7,212	−44,996	10,216

Bayes factor (BF) values represent two times the difference in marginal likelihood estimates between each model and the best‐fit model (“seven‐species”).

a Seven‐species: *P. eburnea* (west), *P. eburnea* (east), *P. xiziae*,* P. lutea*,* P. polycephala*,* P. alutacea*,* P. suichuanensis*; six‐species (a): [*P. eburnea* (west)+ *P. eburnea* (east)], *P. xiziae*,* P. lutea*,* P. polycephala*,* P. alutacea*,* P. suichuanensis*; six‐species (b): *P. eburnea* (west), [*P. eburnea* (east) + *P. xiziae*], *P. lutea*,* P. polycephala*,* P. alutacea*,* P. suichuanensis*; five‐species: *P. eburnea* (west), *P. eburnea* (east), *P. xiziae*,* P. lutea*, (*P. polycephala* + *P. alutacea* + *P. suichuanensis*); and four‐species: *P. eburnea* (west), [*P. eburnea* (east) + *P. xiziae*], *P. lutea*, (*P. polycephala* + *P. alutacea* + *P. suichuanensis*).

### 3.5 Test of hybridization and gene flow

The jml analyses demonstrated that nine comparisons exhibited genetic distances that were significantly lower than expected (*P *<* *.05; Table S7), suggesting that incomplete lineage sorting alone could not explain the discordance across datasets and potential interspecies hybridization might have occurred.

ima2 analyses with the three datasets are summarized in Table 5. For the four‐lineage dataset, significant levels of unidirectional gene flow (2*N*m = 0.037−0.077) were detected between lineages, with the exception for significantly bidirectional gene flow detected between lineages of PAS (*P. polycephala *+ *P. alutacea *+ *P. suichuanensis*) and EX (eastern *P. eburnea *+ *P. xiziae*). Further ima2 runs within lineages revealed significantly unidirectional gene flow from EbuE into Xiz, whereas no significant gene flow was detected among species from PAS.

**Table 5:** Maximum likelihood estimates of population migration rate (2*N*_x_ *M*_x>y_) with the isolation–migration (IMa2) analyses

Species pair (species 0 and species 1)	2*N*_0_ *M*_0>1_	2*N*_1_ *M*_1>0_
Among four lineages		
EbuW and PAS	.002 (0, .245)	.067* (0, .298)
EbuW and Lut	.004 (0, .165)	.037* (0, .204)
EbuW and EX	.049* (0, .204)	.002 (0, .130)
PAS and Lut	.003 (0, .181)	.003 (0, .155)
PAS and EX	.055** (0, .264)	.072* (0, .264)
Lut and EX	.002 (0, .124)	.077* (.009, .285)
Among Pol, Alu and Sui		
Pol and Alu	.260 (0, 1.070)	.168 (0, .744)
Pol and Sui	.002 (0, .362)	.001 (0, .240)
Alu and Sui	.005 (0, .487)	.004 (0, .550)
Between EubE and Xiz		
EbuE and Xiz	.096 (0, .446)	.088* (0, .265)

Values between parentheses represent the range of 95% highest posterior densities of migration rate. 2*N*_x_ *M*_x>y_: the population migration rate from population y to population x. *p < .05 and *^*^ *p* < .01 are statistical significance of likelihood ratio test. Lineage and species abbreviations are defined in Table 1.

Four evolutionary scenarios were modeled to determine the temporal pattern of gene flow. A scenario of historical gene flow (Scenario B; Figure 5) was the most probable model for the four‐lineage dataset; such a model was also highly supported for the three‐lineage dataset. For the two‐lineage dataset, the model of constant gene flow (Scenario C; Figure 5) was slightly higher supported than a scenario of historical gene flow (0.506 vs. 0.494; Table 6).

**Table 6:** Summary results of gene flow model selection based on posterior probabilities under the rejection method using *F*_ST_

Model	Zero	Historical	Constant	Recent
(Lut, (EbuW, (EX, PAS))	0	**.718**	.282	0
(Pol, (Alu, Sui))	0	**.742**	.258	0
(EbuE, Xiz)	0	.494	**.506**	0

The four models of gene flow are illustrated in Figure 2. The model with the highest posterior probability is in bold. Lineage and species abbreviations are defined in Table 1.

The genealogical sorting index (gsi) provides an alternative approach to determine the temporal period of gene flow. Values of gsi for each simulation are listed in Table 7. Our simulations show that gsi values were relatively high in both zero and historical gene flow scenarios (all values ≥0.75; Table 7). The gsi values based on empirical data varied from 0.84 to 1, which strongly matched both the zero and historical gene flow models. However, we cannot differentiate whether models of zero and historical gene flow best explain our empirical results, because of the similar gsi values of the two scenarios.

**Table 7:** Mean values and standard errors (in parentheses) of the genealogical sorting index (gsi) for both empirical and simulated datasets for the four scenarios modeled (see Figure 2)

Species	Empirical	Simulation			
		Zero	Historical	Constant	Recent
EbuW	1	.92 (.05)	.91 (.06)	.38 (.04)	.38 (.04)
EbuE	.95	.93 (.04)	.92 (.05)	.40 (.05)	.40 (.05)
Lut	1	.97 (.05)	.88 (.09)	.31 (.04)	.31 (.04)
Pol	1	.81 (.14)	.81 (.14)	.25 (.04)	.25 (.04)
Alu	.84	.82 (.10)	.82 (.11)	.29 (.04)	.29 (.04)
Sui	.92	.75 (.15)	.75 (.15)	.23 (.04)	.23 (.04)
Xiz	1	.88 (.11)	.87 (.11)	.28 (.04)	.28 (.04)

See Table 1 for species abbreviations.

### 3.6 Differentiation of floral traits

**Figure 6:**
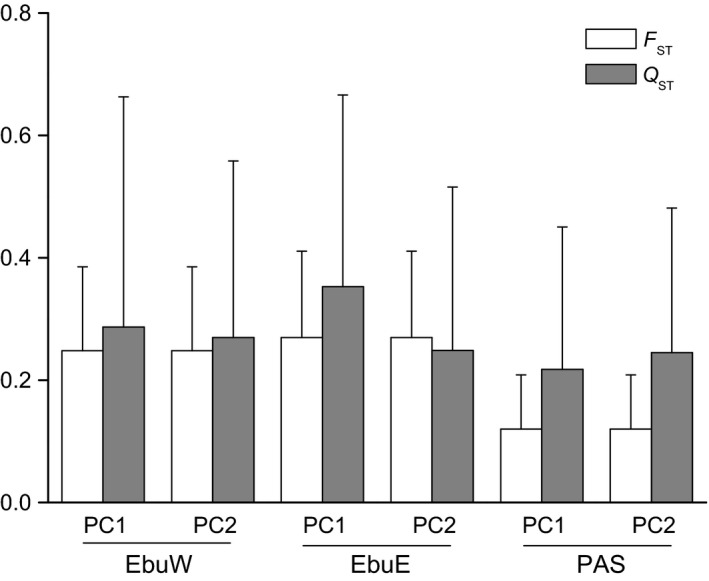
Comparison of *F*_ST_ and *Q*_ST_ values and their 95% confidence intervals. *Q*_ST_ values were calculated from two principal components for the five flower traits. The analyses were performed on three separate datasets: EbuW, EbuE and PAS

We found pronounced differences in the scored flower traits among populations, but none of the *Q*_ST_ estimated differed significantly from SNP‐based *F*_ST_ estimates, with the exception of corolla length (CL) in the eastern populations of *P. eburnea* (Table S8). A principal components analysis of the flower traits found that the first two axes of variation account for most of the variation, with PC1 and PC2 explained 71.2% and 15.6% of total variance, respectively. The *Q*_ST_ values of PC1 and PC2 are similar to that of *F*_ST_ across the three lineages analyzed. Accordingly, no significant difference (overlapping CI in all cases) was observed between the pairwise *Q*_ST_ and neutral genetic variation (*F*_ST_) in all comparisons (Figure 6), suggesting that neutral processes seem to be the most probable cause for divergence in the studied traits.

## 4 DISCUSSION

We conducted a detailed assessment of speciation history of the *P. eburnea* complex from a global biodiversity hot spot through the coupling of multilocus data, phylogenetic reconstruction, coalescent methods and model testing. Our data support the prevalence of allopatric speciation and highlight the role of neutral processes such as genetic drift in the diversification process. At small geographic scales, limited hybridization occurred in the past between proximate populations but did not eliminate species boundaries. We found no evidence of divergent selection contributing to population differentiation of a suite of flower traits.

### 4.1 Phylogenetic relationships, species boundary and genetic differentiation

A previous study based on cpDNA sequences was not able to resolve phylogenetic relationships among taxa of the *P. eburnea* complex (Gao et al., 2015). Based on the coalescent species tree reconstruction of multilocus nuclear DNA sequences, the present study revealed that most recognized species receive strong statistical support. Furthermore, the largely monophyletic relationships of populations within species are evidence that species delimitations are coherent. In addition, the structure analyses of nDNA dataset identified independent lineages with low admixture of genetic composition between species. Individuals from the populations of GXQZ01, JH01, JH03, DX03, NY01 and JXYF01 show substantial mixing in the structure analysis (Fig. S4), which implies that genetic introgression might have played a role in the evolution of these populations (but see below). On the other hand, individuals of the three species of PAS were always inferred to belong to a single genetic cluster for varying values of *K*, suggesting frequent gene flow or incomplete lineage sorting due to recent speciation of the species. Finally, the Neighbor‐Net analyses showed the same overall pattern of genetic relationships among populations and species (Figure 5). Consistent with the species tree and previous cpDNA analyses, our coalescent species delimitation, together with Structure and Neighbor‐Net analyses, resolved the most widespread species *P. eburnea* into two deeply divergent lineages, indicating that the split between the two groups is relatively old, separating two lineages that developed in isolated regions. Nevertheless, nearly all populations were grouped largely according to geographic proximity. These results are in agreement with the strong phylogeographic structure uncovered from previous cpDNA and microsatellite analyses (Gao et al., 2015), suggesting that geographic isolation has played an important role in population and species divergence.

Both nDNA and SNP markers revealed high genetic differentiation among most population‐ and species pairs. Actually, the six nuclear genes revealed a much deeper pattern of population structure than that of 414 SNP markers, suggesting that relatively small numbers of nuclear DNA loci without linkage may be sufficient for detecting population genetic structure when it is pronounced. The finding of high genetic differentiation among populations was consistent with our hypothesis of restricted gene flow and long‐term persistence in geographically isolated karst island landscapes (Gao et al., 2015). As a habitat specialist confined to island‐like karst caves or outcrops, the geographic isolation and small size of populations is expected to lead to reductions in genetic variation as a result of random genetic drift and a decrease in gene flow. Accordingly, most populations show low‐to‐moderate genetic variation (Table S3 and S4). Similar to the scenario in oceanic islands, higher levels of genetic divergence and limited gene flow have been reported for many endemics confined to terrestrial island‐like habitats around the world (Barbará et al., 2007; Byrne & Hopper, 2008; Palma‐Silva et al., 2011; Tapper et al., 2014). In particular, the pattern of genetic variation observed within and between populations of *Primulina* is intriguingly similar to that of *Begonia* (Hughes & Hollingsworth, 2008; Hughes, Hollingsworth, & Miller, 2003). Both genera are characterized by their high levels of local endemism and karst habitat specialization in southern China. Taken together, these results have highlighted the role of isolation and genetic drift in shaping population differentiation in the karst endemics.

### 4.2 Spatial and temporal patterns of gene flow

Despite the pattern of high genetic differentiation observed for within‐ and among species, the structure and SplitsTree analyses provide evidence for considerable admixture in several western *P. eburnea* populations (GXQZ01, DX03, JH01, JH03) that occur along geographic boundaries between species (Figure 1). The discordance of genetic structure between molecular marker types could be explained by incomplete lineage sorting (i.e., the retention of shared ancestral polymorphisms) or alternatively gene flow. Differentiating between the genetic signatures of incomplete lineage sorting and gene flow is difficult; however, distinguishing between the two is important in understanding the speciation history of closely related species. In our case, the incomplete lineage sorting scenario seems to be improbable because the divergence between *P. eburnea* (west) with other lineages is relatively old (ca. 5.52 Ma; Gao et al., 2015), demonstrating that these lineages have been diverged for a long time, allowing complete lineage sorting to occur. Additionally, the incomplete lineage sorting predicts a geographically independent genealogical signature. In our case, both nDNA and SNP dataset analyses revealed strong spatial structure of genetic lineages, with the weakly differentiated populations concentrated in the zone of geographic proximity. Furthermore, the statistical test for incomplete lineage sorting versus hybridization with jml analyses indicated that incomplete lineage sorting alone is insufficient to explain the observed level genetic variation across species with independent genes and species. Overall, these results provide support for a gene flow scenario among species. As expected for the gene flow scenario, the IM analysis suggests significant gene flow between the western *P. eburnea* populations and the three other lineages (Table 5). Furthermore, both gsi and ABC simulations suggest historical rather than contemporary gene flow (e.g., secondary contact) between species. Hence, the most plausible explanations for the genetically mixed populations (GXQZ01, DX03, JH01, JH03) might be the interspecific hybridization events among those taxa happened just after their divergence or at some point in the past, without current hybridization.

Similarly, both Structure and SplitsTree analyses demonstrated a close genetic relationship between *P. xiziae* and the eastern *P. eburnea* populations of NY01 and GY01, which could be a result of significant gene flow from eastern *P. eburnea* lineage into *P. xiziae* as demonstrated in the IM analysis. Consistent with the previous results based on cpDNA and microsatellite analyses, both nDNA loci and SNP markers revealed a shallow genetic divergence between *P. xiziae* and the eastern *P. eburnea* lineage with *F*_ST_ values even lower than most intraspecific comparisons. These results may reflect recent speciation (ca. 1.95 Ma; Gao et al., 2015) between the two species. In fact, *P. xiziae* was formerly recognized as *P. eburnea* and recently classified as a separate species due to its distinctive morphology and phenology (Li et al., 2012). Our ABC‐based model selection procedure resulted in equal support for both constant and historical gene flow scenarios. However, the gsi statistics most strongly supported either no gene flow or historical gene flow scenarios (all values ≥0.87; Table 7). Taking these results together, a scenario of historical gene flow between *P. xiziae* and eastern *P. eburnea* is more likely. However, the interpretation of these results should be cautious because the choice of summary statistics and model selection criteria might influence the conclusions that one draws (e.g., Csilléry, Blum, Gaggiotti, & Francois, 2010).

The lack of genetic differentiation among species of the PAS lineage may be due to their recent divergence or high levels of gene flow. However, IMA2 analysis did not find evidence of significant gene flow among species. This result seems to suggest a scenario of allopatric speciation, where a decline in gene flow could be due simply to the physical isolation between recently diverged species. However, the ABC models support a scenario of historical gene flow rather than a scenario of isolation model with no gene flow. Therefore, this lack of differentiation is most likely a consequence of their recent origin and the high level of shared ancestral polymorphism considerably reducing the power of tests for finding postdivergence gene flow.

Nevertheless, the level of gene flow we detected in all comparisons was far below 0.5 *N*_e_m per generation (Table 5), a value assumed insufficient to counteract random drift (Wright, 1969). The limited gene flow estimated for *Primulina* is in line with the results from the previous cpDNA and microsatellites study, where most populations of the complex were fixed for a single private haplotype, which resulted in a high population differentiation (*F*_ST _= 0.944) (Gao et al., 2015). The high genetic differentiation revealed by multiple nuclear and SNP markers in this study also provides evidence for limited gene flow. Therefore, gene flow, if any, has only a minor impact on population divergence and speciation of *Primulina*.

### 4.3 Speciation history of the *P. eburnea* complex

Based on the strong signature of phylogeographic structure and current nonoverlapping distributions, Gao et al. (2015) argued for an allopatric mode of speciation for the *P. eburnea* complex. In agreement with this viewpoint, the nonsignificant gene flow inferred among species of the lineage PAS supports allopatric divergence without subsequent genetic exchange. In contrast, the significant postdivergence gene flow within the four lineages and between *P. xiziae* and eastern *P. eburnea* is therefore inconsistent with a strictly allopatric speciation mode, which assumes complete termination of gene flow after initial geographic isolation between two species. Furthermore, apparent genetic admixture in several populations (Figure 4; Fig. S3) along range boundaries is evidence for gene flow between species. However, our simulation results showed that interspecies gene flow is a historical event and is not currently ongoing. These results are essentially in congruence with a scenario of parapatric speciation, where genetic introgression occurred at some point in the past between populations with proximate distributions before complete reproduction isolation. As posited above, however, the inferred gene flow for all comparisons was far below 0.5 *N*_e_m per generation, suggesting a minor role of gene flow on speciation. Therefore, allopatric speciation could have been the predominant geographic modes of speciation. Similar to our results, allopatric speciation accompanied by hybridization has been argued as a major evolutionary driver for endemics from the Balkan Peninsula (Olšavská, Slovák, Marhold, Štubňová, & Kučera, 2016).

Few studies have tested the geographic mode of speciation in karst areas; it is believed that the highly heterogeneous topography of the mountain chains in southern China favored the emergence of new lineages (López‐Pujol et al., 2011a, 2011b), which are generally thought to have evolved as a result of microallopatric speciation. Seeds of *Primulina* are small but not small enough for effective wind dispersal and do not have dispersal aids (Li & Wang, 2004). Therefore, we would argue that the geographic isolation and the low dispersal ability in *Primulina* are main driving forces that have promoted genetic isolation among populations, leading to allopatric speciation at local scales, thus representing a case of “terrestrial island” speciation. Recently, allopatric speciation has been proposed as the primary mode responsible for species diversification of *Begonia* (Chung et al., 2014), a genus similar to *Primulina* in that they exhibit extremely high species richness and endemism confined to karst landscapes in southern China and Southeast Asia. A similar scenario has also been suggested for explaining the high species diversity and narrow endemism of several members of New World Gesneriaceae, associated with geographically isolated rock outcrops in southern and southeastern Brazil (Perret, Chautems, Spichiger, Barraclough, & Savolainen, 2007).

Despite speciation with or without gene flow, adaptive differentiation can occur if natural selection is strong enough to cause significant fitness differences across different environment conditions. Floral traits are commonly thought to be a key innovation involved in the diversification of angiosperms. Our field work indicates that the *P. eburnea* complex is pollinated by bees (i.e., *Amegilla* spp., unpublished data). Although we cannot determine whether pollinator shifts have occurred among species, Ellis and Johnson (2009) suggested that floral variation can evolve without pollinator shifts, potentially driven by pleiotropic effects, herbivores, abiotic agents and/or genetic drift in isolated and small populations. In the *P. eburnea* complex, lack of significant difference in the levels of differentiation between flower traits (*Q*_ST_) and neutral genetic markers (*F*_ST_) indicates that genetic drift may have played a major role in the observed pattern of differentiation among species. As a habitat specialist confined to island‐like karst caves or outcrops, population sizes of *Primulina* are usually very small. In fact, both theoretical and empirical studies (Barrett & Schluter, 2007; Fisher, 1930; Woolfit & Bromham, 2005) suggest selection may become less efficient relative to drift in small populations. However, it should be noted that the difference in quantitative traits in this study was only estimated from floral traits. Leaf traits are another group of impotently functional traits, which deserve future investigations.

The high genetic differentiation, low levels of gene flow and lacking evidence of divergent selection on flower traits support the idea that neutral processes, such as genetic drift, have played an important role in driving species diversification in the complex. Although the contribution of genetic drift in speciation has been the subject of keen debate in this century (e.g., Sobel, Chen, Watt, & Schemske, 2010; Turelli, Barton, & Coyne, 2001), several empirical studies demonstrated that divergent selection had likely played little role in population divergence, suggesting that genetic drift might have played a large role in speciation (Boucher, Zimmermann, & Conti, 2016; Comes, Tribsch, & Bittkau, 2008; Kozak, Weisrock, & Larson, 2006; Verboom, Bergh, Haiden, Hoffmann, & Britton, 2015). Drift is especially likely to be an important driver of speciation for species occurring in small and isolated (allopatric) populations from island or island‐like systems, where the small effective population size and limited gene flow are expected to increase the relative importance of drift as an evolutionary process (Templeton, 2008). In *Primulina*, the low genetic diversity and strong population differentiation could be related to small population sizes in highly dissected landscapes and low dispersal abilities, which strengthen the influence of genetic drift.

### 4.4 Conservation implications

Due to its unique natural landforms and the associated special biota, “South China Karst” has been listed as a World Natural Heritage site, which warrants high priority for conservation. Plants restricted to karst island habitats, because of their small population sizes and physical barriers, are particularly sensitive to habitat loss and climate change (Damschen, Harrison, Arckerly, Fernandez‐Going, & Anacker, 2012). The results of this study have obvious conservation implications, not just for the *P. eburnea* species complex, but also other karst endemics in this region. The low levels of genetic variation within populations (*c*.30%) suggest a substantial amount of genetic variation would be lost following even a few local populations. The naturally small population sizes and lack of gene flow make the populations highly vulnerable to extinction due to both demographic and genetic factors. Fine‐scale studies of population connectivity incorporating landscape information will further inform conservation efforts and population management.

## DATA ARCHIVING STATEMENT

Data for this study are available at: https://doi.org/10.5061/dryad.8b69r.
