# Supplementary material for: Speciation history of a species complex of Primulina eburnea (Gesneriaceae) from limestone karsts of southern China, a biodiversity hot spot
Source: Evol Appl. 2017 Jun 22;10(9):919–34. doi: 10.1111/eva.12495 (PMC5680421; doi:10.1111/eva.12495)
Supplement: Supplementary file 1 [file EVA-10-919-s001.docx]

**Fig. S1** Bayesian gene trees for the *Primulina eburnea* complex based on haplotypes of six nuclear genes (*7FR, 13FR, 97FR, 117FR, 155FR and 248FR*). Species abbreviations are defined in Table 1.


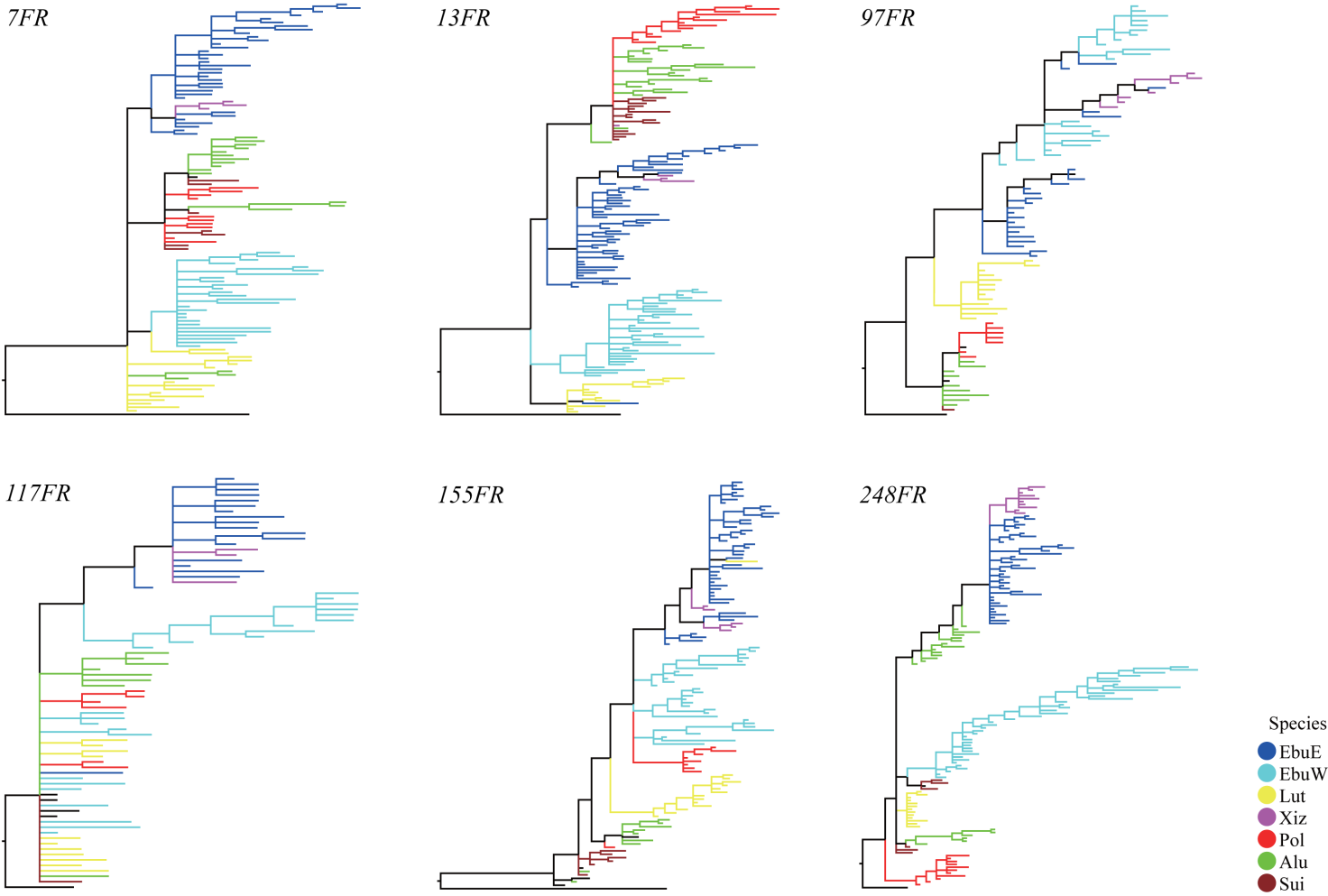


**Fig. S2** Plots of ΔK for each K for (a)nuclear DNA sequences and (b) SNP data sets according to Evanno et al. (2005).

(a) (b)


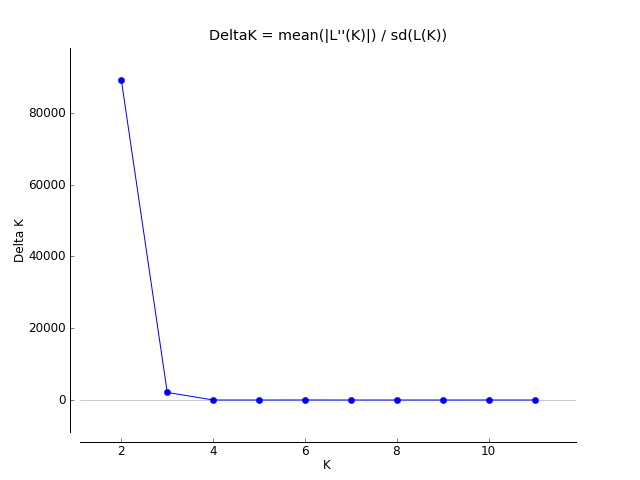

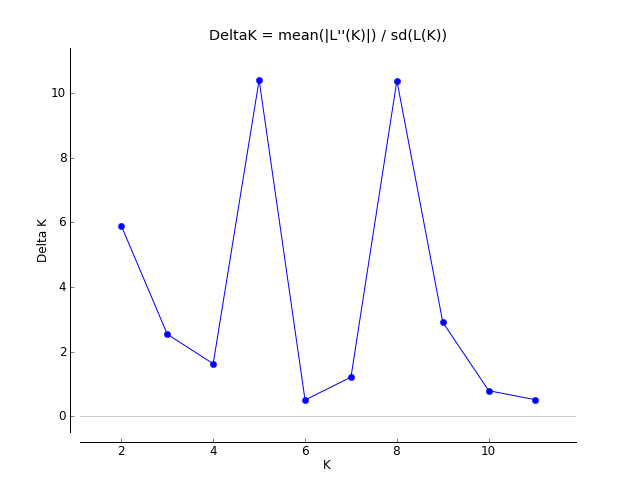


**Fig. S3** Bayesian assignment of 384 individuals using Structure with *K* from 2 to 4. Each individual is represented by a vertical bar and grouped by population and species. Population names correspond to Table S1, Supporting information.

**
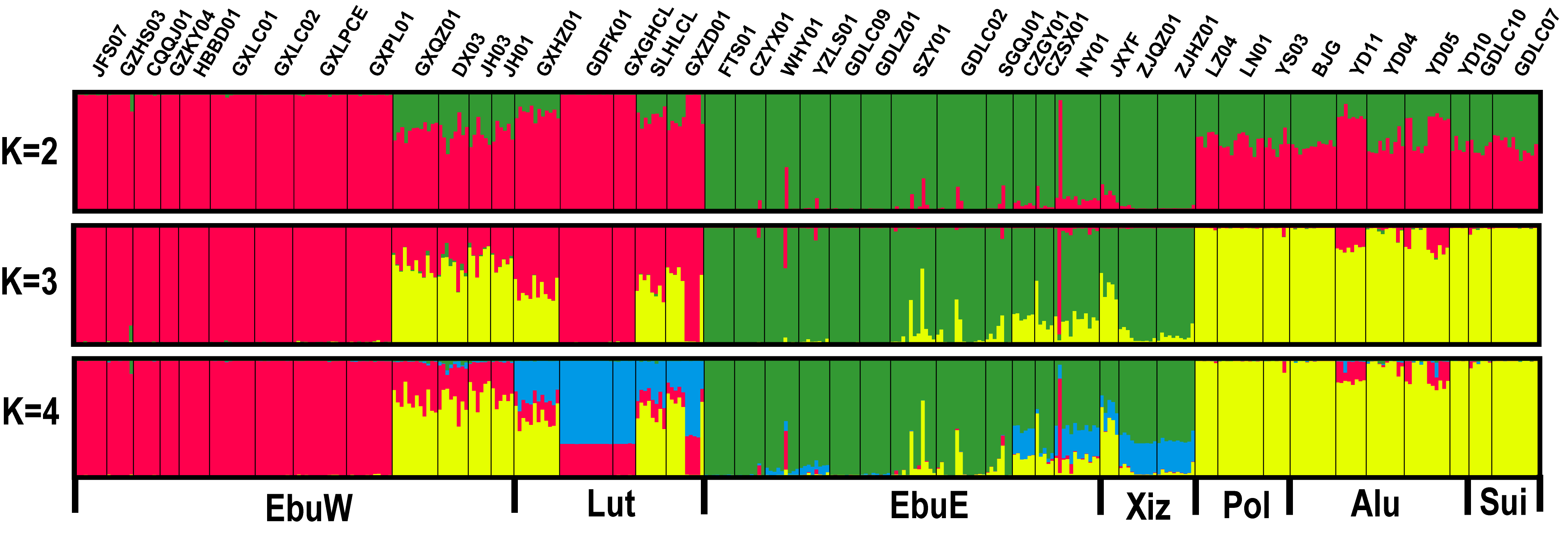
**
