# Supplementary figures and images for: Speciation history of a species complex of Primulina eburnea (Gesneriaceae) from limestone karsts of southern China, a biodiversity hot spot
Source: Evol Appl. 2017 Jun 22;10(9):919–34. doi: 10.1111/eva.12495 (PMC5680421; doi:10.1111/eva.12495)

- (A)
- *P. eburnea* (West)
  - *P. eburnea* (East)
  - *P. lutea*
  - *P. polycephala*
  - *P. alutacea*
  - *P. suichuanensis*
  - *P. xizae*

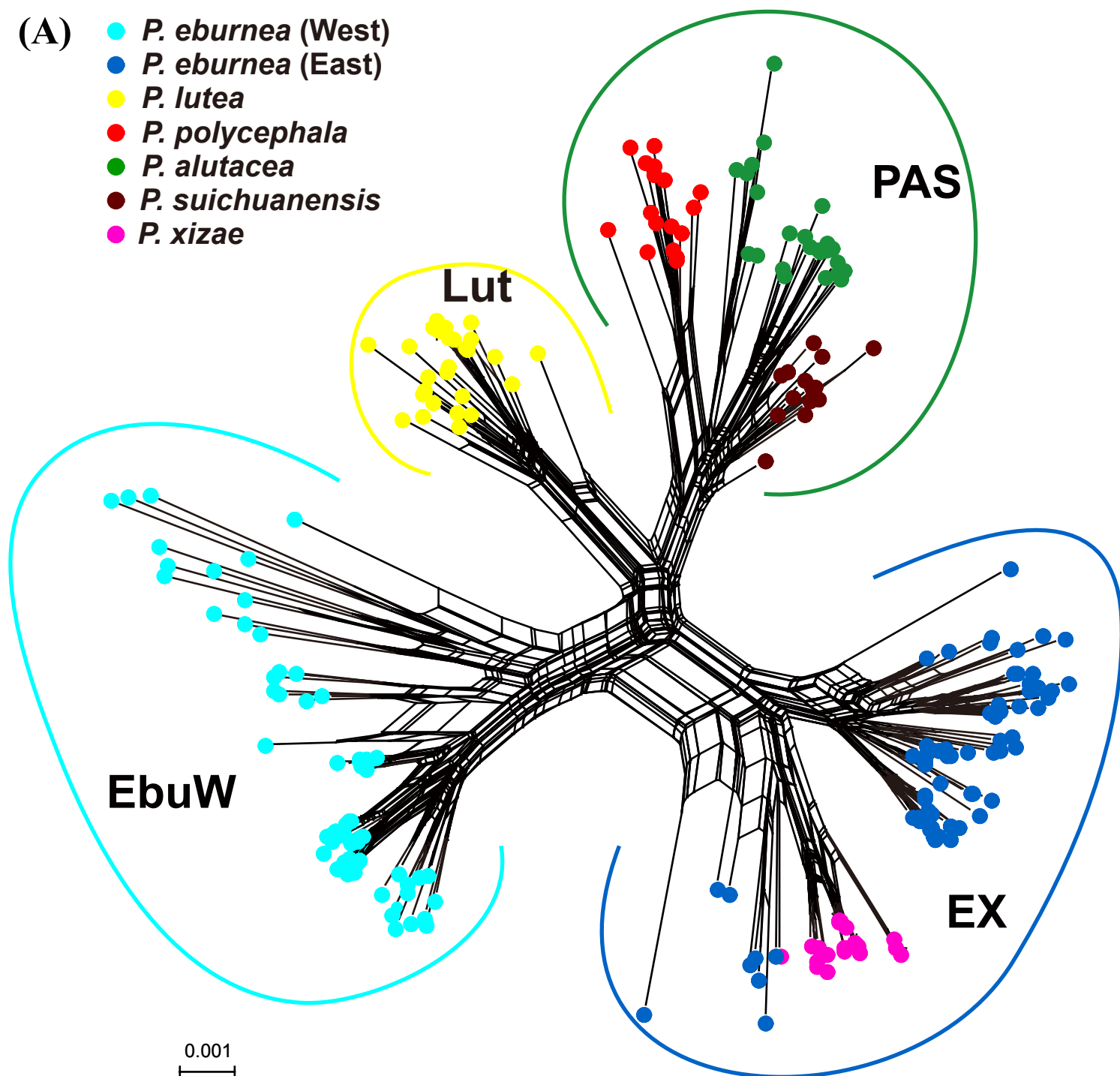

(B)

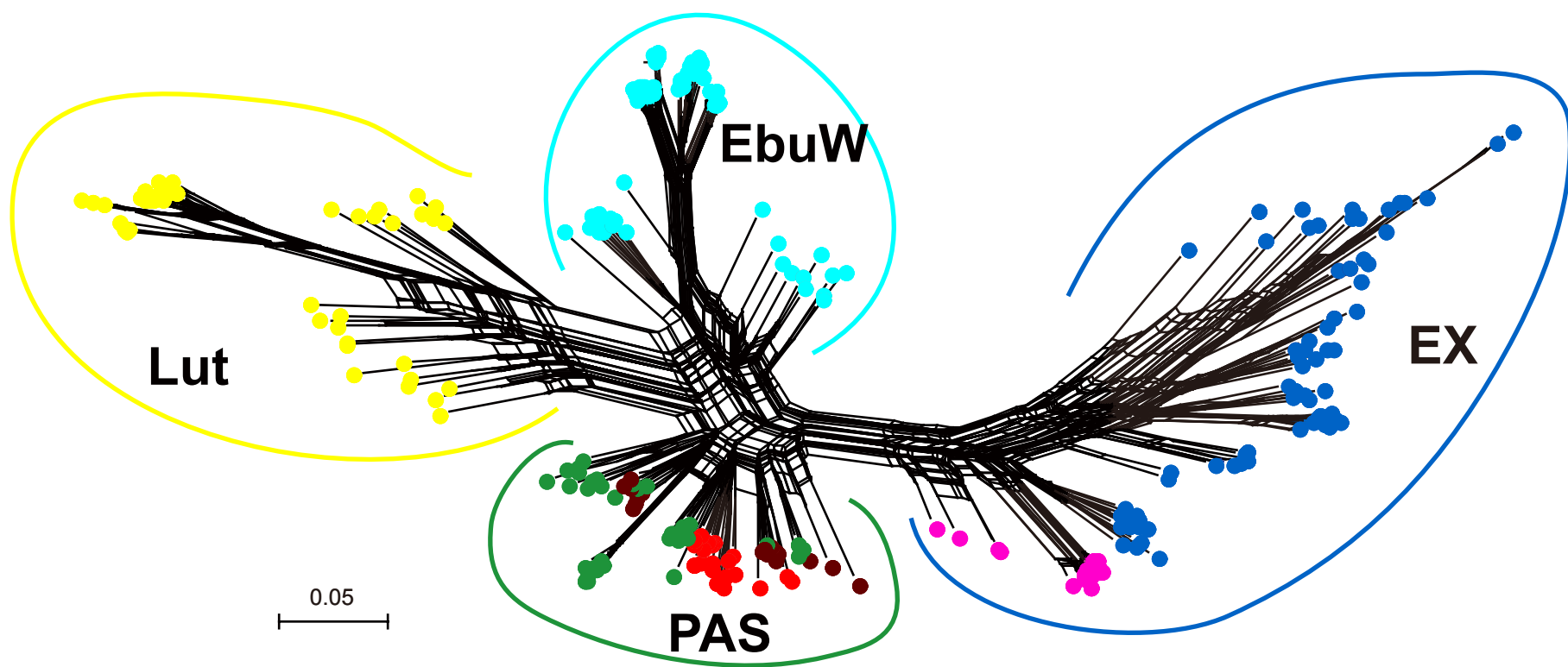

Supplement: Supplementary file 2 [file EVA-10-919-s002.xlsx]
